# Pharmacological inhibitors of anaplastic lymphoma kinase (ALK) induce immunogenic cell death through on-target effects

**DOI:** 10.1038/s41419-021-03997-x

**Published:** 2021-07-16

**Authors:** Adriana Petrazzuolo, Maria Perez-Lanzon, Isabelle Martins, Peng Liu, Oliver Kepp, Véronique Minard-Colin, Maria Chiara Maiuri, Guido Kroemer

**Affiliations:** 1 Centre de Recherche des Cordeliers, INSERM UMRS1138, Université de Paris, Sorbonne Université, Team “Metabolism, Cancer & Immunity”, 75006 Paris, France; 2 Cell Biology and Metabolomics platforms, Gustave Roussy Cancer Campus, 94805 Villejuif, France; 3 University Paris Saclay, Faculty of Medicine, 94270 Kremlin Bicêtre, France; 4 Department of Pediatric and Adolescent Oncology, Gustave Roussy, Université Paris-Saclay, 94805 Villejuif, France; 5 Pôle de Biologie, Hôpital Européen Georges Pompidou, AP-HP, 75015 Paris, France; 6 Suzhou Institute for Systems Medicine, Chinese Academy of Sciences, Suzhou, Jiangsu 215163 China; 7 Karolinska Institute, Department of Women’s and Children’s Health, Karolinska University Hospital, 17176 Stockholm, Sweden

**Keywords:** Cancer, Cancer models

## Abstract

Immunogenic cell death (ICD) is clinically relevant because cytotoxicants that kill malignant cells via ICD elicit anticancer immune responses that prolong the effects of chemotherapies beyond treatment discontinuation. ICD is characterized by a series of stereotyped changes that increase the immunogenicity of dying cells: exposure of calreticulin on the cell surface, release of ATP and high mobility group box 1 protein, as well as a type I interferon response. Here, we examined the possibility that inhibition of an oncogenic kinase, anaplastic lymphoma kinase (ALK), might trigger ICD in anaplastic large cell lymphoma (ALCL) in which ALK is activated due to a chromosomal translocation. Multiple lines of evidence plead in favor of specific ICD-inducing effects of crizotinib and ceritinib in ALK-dependent ALCL: (i) they induce ICD stigmata at pharmacologically relevant, low concentrations; (ii) can be mimicked in their ICD-inducing effects by ALK knockdown; (iii) lose their effects in the context of resistance-conferring ALK mutants; (iv) ICD-inducing effects are mimicked by inhibition of the signal transduction pathways operating downstream of ALK. When ceritinib-treated murine ALK-expressing ALCL cells were inoculated into the left flank of immunocompetent syngeneic mice, they induced an immune response that slowed down the growth of live ALCL cells implanted in the right flank. Although ceritinib induced a transient shrinkage of tumors in lymphoma-bearing mice, irrespective of their immunocompetence, relapses occurred more frequently in the context of immunodeficiency, reducing the effects of ceritinib on survival by approximately 50%. Complete cure only occurred in immunocompetent mice and conferred protection to rechallenge with the same ALK-expressing lymphoma but not with another unrelated lymphoma. Moreover, immunotherapy with PD-1 blockade tended to increase cure rates. Altogether, these results support the contention that specific ALK inhibition stimulates the immune system by inducing ICD in ALK-positive ALCL.

## Introduction

Cancer has been conceived as an essentially cell autonomous disease (caused by [epi]genetically unstable cancer cells that proliferate, invade and disseminate) [1] until it became clear that cancer cells also need to elude immune control to generate invasive tumors [2]. Indeed, in normal tissue homeostasis, aberrant cells are eliminated by innate immune effectors (such as NK cells) or even elicit cognate immune responses (mostly by cytotoxic T lymphocytes) [3, 4]. In contrast, immunosuppression governs invasive tumors. Genetic tumor aberrations guide immune escape, recruiting immunosuppressive macrophages or myeloid-derived suppressor cells [5, 6] or hiding tumor cells from recognition (downregulating class I major histocompatibility complex) [7].

In this context, an authentic paradigm shift has occurred over the past decade. Beyond the idea that anticancer drugs must kill or paralyze the malignant cells themselves, an ever-increasing armamentarium of immuno-oncology drugs is being developed to stimulate (or unblock) immune responses against tumor-associated antigens [8]. For example, immune checkpoint inhibitors that target the immunosuppressive PD-1/PD-L1 interaction are being approved for ever more oncological indications, across a wide spectrum of malignant disease [9–12].

Curiously, it turned out that many (relatively) successful anticancer chemotherapeutics do not only kill neoplastic cells but rather succeed in inducing potent and clinically relevant antitumor immune responses. Often, such effects are obtained through the induction of immunogenic cell death (ICD), a cell death modality that arises in the context of apoptosis [13], necroptosis [14], pyroptosis [15], or ferroptosis [16]. Apoptotic (caspase-dependent) ICD is characterized by a series of stereotyped changes in which danger-associated molecular patterns (DAMPs) are exposed on the cell surface or released into the extracellular space. Cell surface-exposed DAMPs include calreticulin (CALR), which usually is present in the lumen of the endoplasmic reticulum, yet translocates during ICD to the external side of the plasma membrane and then acts as a potent ‘eat-me’ signal for dendritic cells (DCs) [17]. Released DAMPs include adenosine triphosphate (ATP), which is a chemoattractant for DC precursors [18], high mobility group box 1 (HMGB1), which is a maturation/activation factor for DCs [19], and type I interferons that attract T lymphocytes into the tumor bed [20]. Altogether, a spatiotemporal sequence of DAMPs orchestrates the recruitment of DCs into the tumor, the uptake of tumor antigens by DCs, and the cross-presentation of such antigens to cytotoxic T lymphocytes. The latter then launch an attack against residual cancer cells and are responsible for the long-term outcome of ICD-inducing chemotherapies beyond treatment discontinuation.

ICD has become a therapeutically important cell death modality because some ICD-relevant biomarkers inform on the prognosis of cancer patients. For example, the loss of CALR expression by cancer cells correlates with dismal prognosis in acute myeloid leukemia [21], colorectal cancer [22], non-small cell lung cancer [23], and ovarian carcinoma [24]. The absence of extracellular ATP (e.g., due to the overexpression of ATP-degrading enzymes), the suppression of HMGB1 expression and the subversion of type I interferon responses also have a negative prognostic impact [25–27].

The idea that anticancer agents can ignite therapeutically relevant antitumor immune responses has been extended to so-called ‘targeted’ agents, hitting genetic abnormalities unique to cancer cells [28, 29]. Thus, the first tyrosine kinase inhibitor (TKI) that was introduced into the clinic, imatinib mesylate [30], an inhibitor of oncogenic breakpoint cluster region-abelson tyrosine protein kinase, BCR-ABL, and C-KIT, turned out to exert (part of) its activity against gastrointestinal stromal tumors (which depend on oncogenic C-KIT) by harnessing the immune system [31–33]. Imatinib mesylate negatively regulates indoleamine 2,3-dioxygenase expression in tumor cells, reducing immunosuppressive tryptophan metabolites in the tumor bed, hence increasing cytolytic T lymphocytes influx and activation [34]. In addition, a recent screen for TKI with ICD-inducing properties led to the identification of crizotinib (used for the treatment of tumors that depend on anaplastic lymphoma kinase, ALK, mesenchymal–epithelial transition factor, c-MET or ROS1), as an ICD-inducing agent, though through off-target effects [35]. Thus, crizotinib induced ICD in cells that lacked the oncogenic kinases that it normally inhibits, and only at doses ≥10 µM, far above the threshold of specificity [35].

Here, we examined the possible on-target effects of crizotinib as TKI specifically inhibiting ALK, using ALK-positive anaplastic large cell lymphoma (ALK^+^ ALCL) as a model, where ALK is expressed and activated following a chromosomal translocation (t(2;5)) leading to the fusion with nucleophosmin 1 (NPM1) [36]. Hence, we investigated the possible pro-ICD and immunogenic effects of ALK kinase inhibitors on an ALK-dependent cancer. We show that such TKIs indeed can trigger ICD through on-target effects.

## Results

### ALK inhibitors induce immunogenic cell death in vitro

**Fig. 1:**
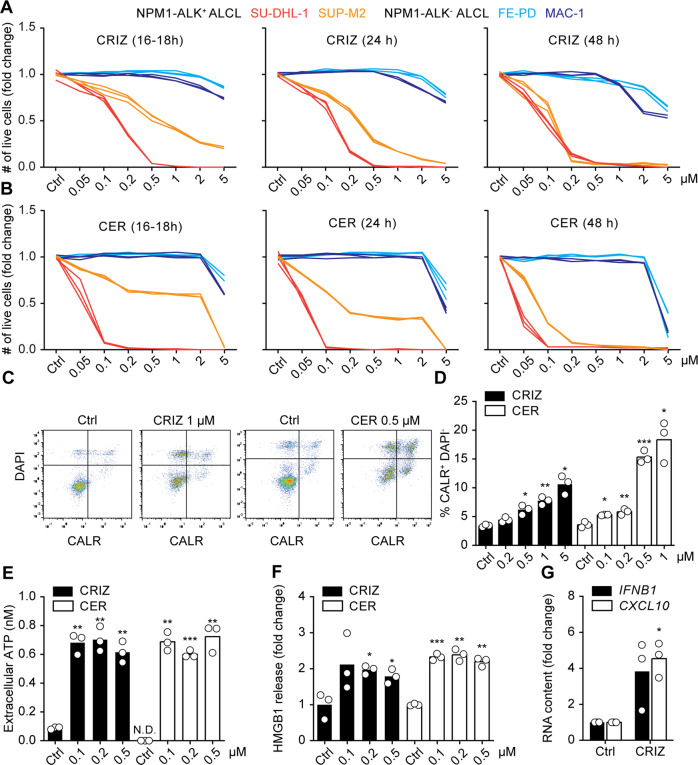
Crizotinib- and ceritinib-induced immunogenic cell death. Human NPM1-ALK-positive (NPM1-ALK^+^) SU-DHL-1 and SUP-M2 cells and the NPM1-ALK-negative (NPM1-ALK^-^) FE-PD and MAC-1 cells were treated with increasing concentrations of crizotinib (CRIZ) (A) or ceritinib (CER) (B) for 16–18, 24, or 48 h. Cell death was evaluated using the mitochondrial transmembrane potential-sensible dye 3,3′-dihexyloxacarbocyanine iodide (DiOC_6_(3)) and 4′,6-diamidino-2-phenylindole (DAPI). The number of live cells (DiOC_6_(3)^+^ DAPI^-^) normalized to vehicle control is represented for each replicate (*n* = 3) (**A**, **B**). NPM1-ALK^+^ SU-DHL-1 cells were treated with CRIZ or CER at the indicated concentrations, for 6 h, and calreticulin (CALR) exposed on the cell surface was quantified by flow cytometry using an indirect immunofluorescence staining. DAPI was used to exclude permeabilized cells. A representative gating strategy is shown in **C,** and the percentage of CALR^+^ DAPI^-^ cells is depicted in **D** (*n* = 3). NPM1-ALK^+^ SU-DHL-1 cells were treated with CRIZ or CER for 24 h and ATP secreted in the extracellular milieu was quantified using a luciferase-dependent assay. Individual values are depicted in **E** (*n* = 3). NPM1-ALK^+^ SU-DHL-1 cells were treated with CRIZ or CER for 48 h and high mobility group box 1 (HMGB1) was quantified in the supernatant using a specific ELISA. Fold increase for each replicate is shown in **F** (*n* = 3). One representative out of three independent experiments is shown **A**–**F**. NPM1-ALK^+^ SU-DHL-1 cells were treated with 0.1 μM of CRIZ for 16–18 h. *IFNB1* and *CXCL10* upregulation was assessed by quantitative PCR using specific fluorescently labeled primer–probe sets. *GAPDH* was used as housekeeping gene. Fold changes of three independent experiments are shown (**G**). Statistical significance was calculated using the Student’s *t* test. *p < 0.05, **p < 0.01, ***p < 0.001 vs. vehicle-treated cells (**D**–**G**). N.D. non-detectable.

NPM1-ALK-positive (NPM1-ALK^+^) tumors rely on ALK kinase activity for survival and proliferation. Thus, ALK inhibition causes tumor cells to halt proliferation and die [37]. We quantified cell death induced by ALK inhibitors using a cytofluorometric approach. This method measures two major features of cell death: the initial loss of mitochondrial transmembrane potential (ΔΨ_m_) and the subsequent loss of the plasma membrane integrity, using 3,3′-dihexyloxacarbocyanine iodide (DiOC_6_(3)) and 4′,6-diamidino-2-phenylindole (DAPI) as fluorescent dyes, respectively. Over the course of cell death, as ΔΨ_m_ is lost, DiOC_6_(3) staining intensity decreases; whereas loss of membrane integrity facilitates DAPI staining. Crizotinib and ceritinib, which are first and second generation ALK inhibitors, respectively, reduced the number of live NPM1-ALK^+^ ALCL cells (DiOC_6_(3)^+^ DAPI^-^), triggering cell death in a dose- and time-dependent manner (Fig. 1A, B, S1). Cell viability was partially rescued by the pan-caspase inhibitor Q-VD-OPh, implying that crizotinib- and ceritinib-induced cell death was apoptotic (caspase-dependent) (Fig. S2). On the contrary, the NPM1-ALK-negative (NPM1-ALK^-^) FE-PD and MAC-1 cells were insensitive to ALK inhibitors, which caused cell death only at the highest concentration tested (5 μM) and later in time (48 h) when compared to NPM1-ALK^+^ cells (Fig. 1A, B, S1). Thus, low concentrations of ALK inhibitors (≤2 µM) have specific effects on NPM1-ALK^+^ but not NPM1-ALK^-^ ALCL cells.

We proceeded exploring the potential immunogenicity of crizotinib- and ceritinib-induced cell death, testing their capability to trigger the surrogate markers of ICD. First, when added to SU-DHL-1 (Fig. 1C–G) or SUP-M2 (Fig. S3) cells, crizotinib and ceritinib caused CALR translocation from the endoplasmic reticulum to the plasma membrane (Fig. 1C, D, S3A, B). Moreover, both inhibitors significantly induced ATP secretion (Fig. 1E, S3C) and HMGB1 release (Fig. 1F, S3D) into the extracellular space. Finally, crizotinib upregulated two pivotal genes of the type I interferon signature, *interferon beta 1* (*IFNB1*) and *C-X-C motif chemokine ligand 10* (*CXCL10*) at the mRNA level (Fig. 1G, S3E). Altogether, our data suggest that crizotinib and ceritinib cause immunogenic cell death of ALK-dependent tumors.

### Specific ALK inhibition causes immunogenic cell death

**Fig. 2:**
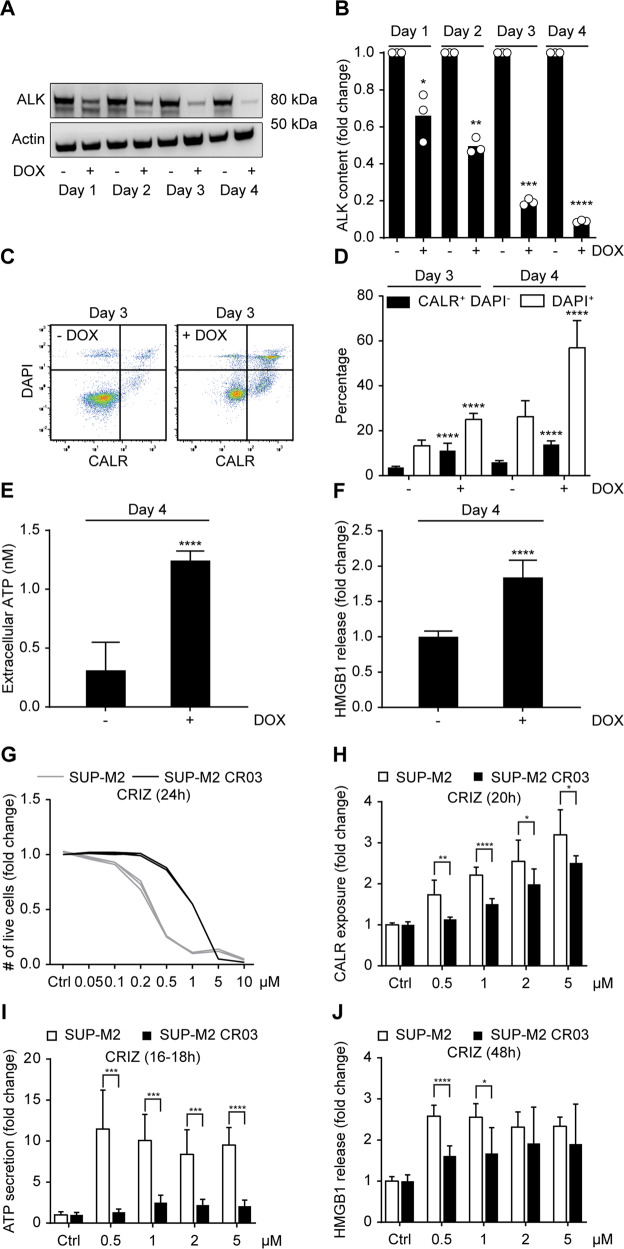
Specific ALK inhibition induces immunogenic cell death. Immunogenic cell death of human NPM1-ALK^+^ SU-DHL-1 TTA cell line expressing a doxycycline-inducible shRNA against ALK (**A**–**F**). shRNA induction was achieved after treatment with 1 μg/mL of doxycycline. ALK protein downregulation was confirmed by Western blot after 1, 2, 3, or 4 days of shRNA induction. Actin was used as loading control. Images are representative of one out of three experiments (**A**). Relative quantification of three independent experiments is shown in **B**. SU-DHL-1 TTA cells were treated with 1 μg/mL of doxycycline for 3 or 4 days and cell death (% DAPI^+^ cells) as well as CALR exposed on the plasma membrane were quantified by flow cytometry. Gating strategy is shown in **C** and means ± SD of 4 independent experiments (*n* = 12) are depicted in **D**. After 4 days of treatment, ATP secretion (**E**) and HMGB1 release (**F**) were quantified with a luciferase-based assay and ELISA, respectively. Means ± SD of 3 independent experiments are shown (*n* = 9). Statistical significance was calculated using the Student’s *t* test. *p < 0.05, **p < 0.01, ***p < 0.001, ****p < 0.0001 vs. vehicle-treated cells (**B**, **D**–**F**). Evaluation of the immunogenic cell death hallmarks in human NPM1-ALK^+^ cell line resistant to crizotinib (**G**–**J**). Crizotinib-resistant (SUP-M2 CR03) and parental (SUP-M2) cells were treated with increasing concentrations of crizotinib (CRIZ) for 24 h and cell death was evaluated using DiOC_6_(3), which labels only mitochondria of live cells, and DAPI entering cells only after plasma membrane permeabilization. The number of live cells (DiOC_6_(3)^+^ DAPI^-^) normalized to vehicle controls is represented for each replicate (*n* = 3) (**G**). CALR translocated to the plasma membrane was quantified by immunofluorescence and flow cytometry after treatment with CRIZ for 20 h. Fold increases were calculated using the median of fluorescent intensity (MFI) of DAPI^-^ cells (**H**). ATP (**I**) and HMGB1 (**J**) released after 16–18 or 48 h of treatment, respectively, were quantified using a luciferase-based assay or an ELISA test. Means of three independent experiments (each made in triplicates) ± SD are shown. Statistical significance was calculated using the multiple Student’s *t* test (*n* = 9); *p < 0.05, **p < 0.01, ***p < 0.001, ****p < 0.0001 (**H**–**J**).

Crizotinib and ceritinib inhibit ALK, as well as other tyrosine kinases (such as ROS1, MET, or Insulin Like Growth Factor 1 Receptor) [38]. Therefore, we attempted to rule out off-target effects of these ALK inhibitors, using two different approaches. In a first set of experiments, we used a human NPM1-ALK^+^ ALCL cell line (SU-DHL-1 TTA) expressing a doxycycline-inducible shRNA against ALK [39]. After doxycycline addition, SU-DHL-1 TTA cells downregulated ALK, halted proliferation and finally died (Fig. 2A–D). In parallel, they externalized CALR (Fig. 2C, D), secreted ATP and released HMGB1 (Fig. 2E, F) into the extracellular milieu. Thus, genetic (non-pharmacological) inhibition of ALK had similar effects as low-doses crizotinib and ceritinib.

In a second set of experiments, we took advantage of an NPM1-ALK^+^ cell line resistant to crizotinib (SUP-M2 CR03) because of a point mutation in the ALK kinase domain that impairs crizotinib binding [40]. As expected, SUP-M2 CR03 resisted crizotinib-induced cell death compared to the parental cell line (Fig. 2G). Concomitantly, a trend for these crizotinib-resistant cells to show less CALR on their surface and release less ATP and HMGB1 than the parental cell line upon challenge with the TKI was clear (Fig. 2H–J). Hence, an ALK mutation that confers resistance to crizotinib-induced cell death also attenuated the stigmata of ICD.

Altogether, these data strongly argue in favor of direct on-target effects of ALK inhibitors with respect to the induction of the surrogate markers of ICD.

### Pathways downstream of ALK mediate immunogenic cell death

**Fig. 3:**
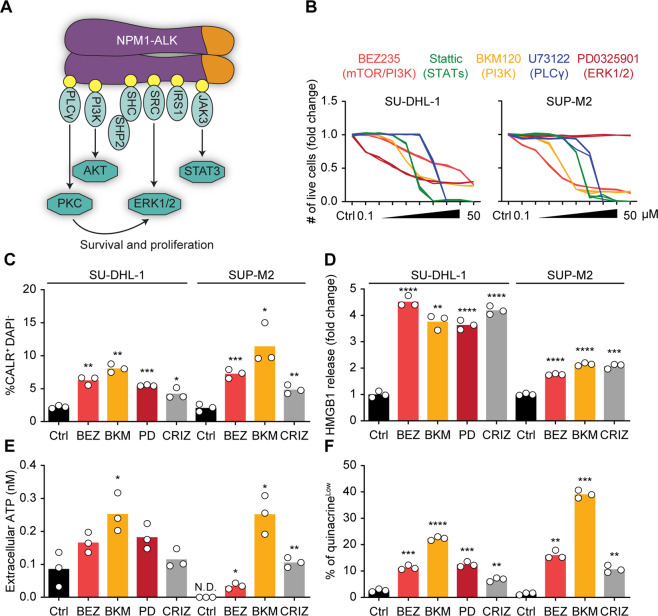
Dissection of ALK downstream pathways inducing immunogenic cell death. Scheme of ALK downstream signaling pathways (**A**). Human NPM1-ALK^+^ SU-DHL-1 and SUP-M2 cells were treated with PI3K, PI3K/mTOR, ERK1/2, PLCɣ, and STATs inhibitors (BEZ235, BKM120, PD0325901, U73122, and stattic, respectively) at 0.1, 0.2, 0.5, 1, 2, 5, 10, 20, and 50 μM. Cell death was evaluated using DiOC_6_(3) and DAPI after 24 h. The number of live cells (DiOC_6_(3)^+^ DAPI^-^), normalized to vehicle control, is shown for each replicate (*n* = 3) (**B**). NPM1-ALK^+^ SU-DHL-1 and SUP-M2 cells were treated with 2 or 5 μM of BEZ235 (BEZ); 2 μM of BKM120 (BKM); 1 or 2 μM of PD0325901 (PD); 0.1 or 0.5 μM of crizotinib (CRIZ). After 16–18 h (SU-DHL-1) or 24 h (SUP-M2) CALR was indirectly stained on the plasma membrane and percentage of CALR^+^ DAPI^-^ cells was quantified by flow cytometry (**C**). ATP (**E**) and HMGB1 (**D**) were quantified in the extracellular milieu of cells treated for 16–18 h (**E**) or 24 h (**D**), using a luciferase-dependent assay or an ELISA, respectively. The propensity of quinacrine to accumulate in ATP-rich vesicles was exploited to quantify the intracellular content of ATP by flow cytometry after 16–18 h of treatment. DAPI was employed as exclusion dye for permeabilized cells. Percentages of quinacrine^low^ cells within DAPI^-^ population are displayed in **F**. Individual replicates are shown (*n* = 3) from one out of three independent experiments. Statistical significance was calculated using the Student’s *t* test. *p < 0.05, **p < 0.01, ***p < 0.001, ****p < 0.0001 *vs*. vehicle-treated cells (**B**–**F**). N.D. non-detectable.

Constitutively active ALK is a clinically relevant oncogene because it ignites several downstream molecular pathways, driving malignant transformation and promoting tumor cell survival and proliferation [41] (Fig. 3A). We wondered which molecular pathway might account for ICD. Therefore, we pharmacologically inhibited each pathway by using inhibitors of PI3K/mTOR, PI3K, ERK1/2, STATs, and PLCγ (BEZ235, BKM120, PD0325901, Stattic, and U73122, respectively). All these pharmacological inhibitors killed SU-DHL-1 cells, whereas SUP-M2 cells proved to be resistant to the ERK1/2 inhibitor PD0325901, as previously reported [42] (Fig. 3B). However, only BEZ235, BKM120, PD0325901 induced death of SU-DHL-1 cells, and only BEZ235 and BKM120 killed SUP-M2 cells in a dose-dependent manner. Hence, we only used these inhibitors to assess the ICD hallmarks (Fig. 3B–F). Moreover, we chose concentrations that yielded comparable levels of cell death. Inhibition of PI3K, PI3K/mTOR, or ERK1/2 activated the molecular pathways leading to CALR translocation to the plasma membrane surface (Fig. 3C, Fig. S4). In addition, BEZ235, BKM120, and PD0325901 provoked HMGB1 and ATP accumulation in the extracellular milieu (Fig. 3D, E). We used an alternative method, quinacrine orange staining, to confirm ATP secretion. Quinacrine accumulates in ATP-rich intracellular vesicles. Thus, ATP secretion coincides with a decrease in quinacrine fluorescence intensity [43]. This method confirmed that inhibition of PI3K, mTOR, or ERK1/2 induced a reduction in cellular ATP content (Fig. 3F, Fig. S5). Among the distinct inhibitors, BKM120 (a PI3K inhibitor) appeared to be the most efficient in inducing signs of ICD (Fig. 3C–F). Two additional PI3K inhibitors, BYL719 and XL147, also killed SU-DHL-1 and SUP-M2 cells and induced the hallmarks of ICD (Fig. S6).

In conclusion, it appears that inhibitors of several pathways operating downstream of ALK mimic the ICD-inducing effects of crizotinib, and among these pathways, PI3K stands out at as a particularly important ICD repressor.

### Immune-dependent effects of ALK-inhibitors in vivo

**Fig. 4:**
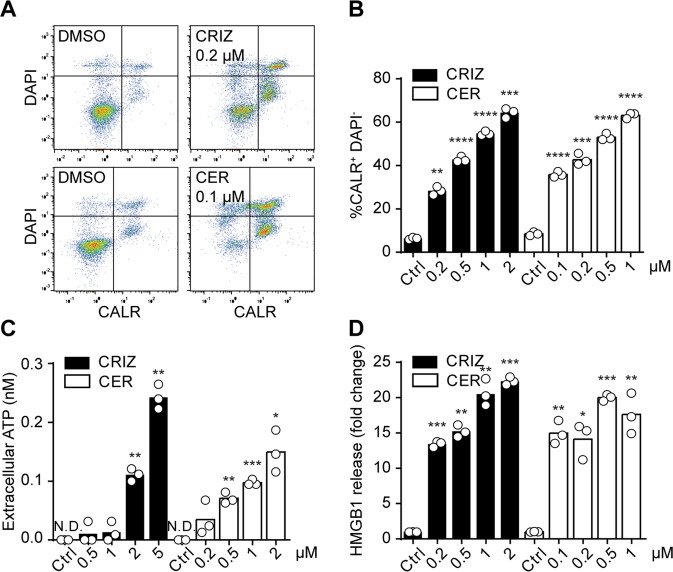
Immunogenic cell death of murine ALK-positive cells. Murine NPM1-ALK^+^ R80 cells were treated with increasing concentrations of crizotinib (CRIZ) or ceritinib (CER) for 6 h. Twenty hours later, exposed CALR was quantified on the surface of DAPI^-^ cells by flow cytometry using an indirect fluorescent staining. Representative dot plots are shown in **A** and percentage of CALR^+^ DAPI^-^ in **B**. ATP (**C**) or HMGB1 (**D**) released in the extracellular milieu of murine NPM1-ALK^+^ R80 cells, treated with CRIZ or CER, were assessed using a luciferase-based assay or an ELISA after 6 or 24 h, respectively. Values of one representative experiment out of three are shown (*n* = 3) (**A**–**D**). Statistical significance was calculated using the Student’s *t* test. *p < 0.05, **p < 0.01, ***p < 0.001, ****p < 0.0001 vs. vehicle-treated cells. N.D. non-detectable.

**Fig. 5:**
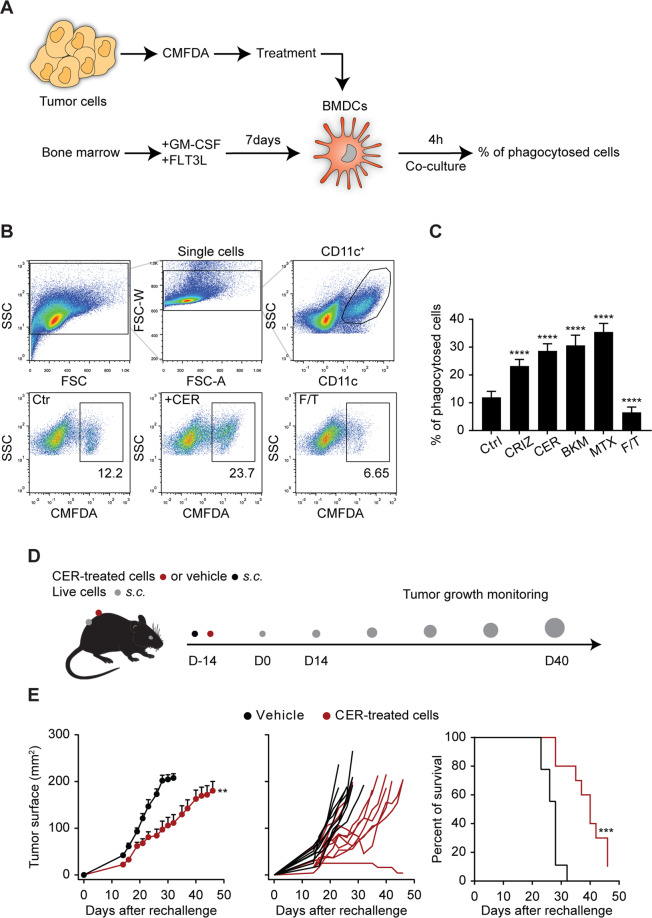
ALK inhibition activates the immune system. As illustrated in the phagocytosis assay scheme (**A**), murine tumor cells were, first, stained with the green cell tracker (CMFDA), then, treated with different drugs, while bone marrow-derived dendritic cells (BMDCs) were isolated from syngeneic mice and differentiated in vitro. After co-culture, the percentages of phagocytized cells were determined by flow cytometry. **B** Murine NPM1-ALK^+^ R80 cells were treated with 0.2 μM of crizotinib (CRIZ) or ceritinib (CER), 2 μM of BKM120 (BKM), 1 μM of mitoxantrone (MTX) for 16–18 h or subjected to one cycle of freeze–thawing (F/T) and co-cultured with BMDCs for 4 h. Co-culture was stained using a fluorescent anti-CD11c antibody. The percentages of CMFDA^+^ cells within CD11c^+^ BMDCs reflect phagocytosis. The representative gating strategy is shown in **B**. **C** Means ± SD of three independent experiments each performed in triplicates (*n* = 9); ****p < 0.0001 (Student’s *t* test) (**C**). Scheme of vaccination experiment (**D**). Murine tumor cells were treated in vitro to reach 50–70% of mortality before being injected into the left flank of syngeneic mice. Two weeks later, mice were challenged with live cells into the opposite flank and tumor appearance and growth were monitored. Five million of NPM1-ALK^+^ R80 cells, previously treated for 24 h with 0.5 μM of CER, were injected into the left flank of C57BL/6 mice. After two weeks, mice were challenged with 1 × 10^6^ of live R80 cells into the opposite flank. Tumor surface in function of time is represented as mean ± SEM of mice belonging to the same group or for each mouse (**E**). Survival is also represented in **E**. Vehicle *n* = 9 and CER *n* = 10. Comparison between tumor growth curves was assessed using a type II ANOVA test, while the statistical significance of survival data was calculated using the Log-rank test. **p < 0.01, ***p < 0.001.

Next, we characterized the immunogenicity of ALK inhibitors in vivo. For that purpose, we first validated the induction of surrogate ICD markers in a murine cell line, R80, generated from transgenic C57BL/6 mice expressing human NPM1-ALK. R80 cells showed signs of ICD after ALK inhibition by crizotinib or ceritinib (Fig. 4). Moreover, when cells were exposed to crizotinib or ceritinib and then co-cultured with bone marrow-derived dendritic cells (BMDCs), clear evidence was found that they were engulfed by BMDCs (Fig. 5A–C) and induced BMDC maturation (Fig. S7).

A standard in vivo assay for ICD consists in the subcutaneous injection of dying/dead cells into immunocompetent mice, followed by rechallenge with live tumor cells 1–2 weeks later into the opposite flank to interpret the reduction of tumor growth as an indication of successful anticancer vaccination [44]. R80 cells were treated with ceritinib in vitro, washed, and then injected subcutaneously (s.c.) into immunocompetent syngeneic C57BL/6. After two weeks, these animals were re-challenged with live R80 injected into the opposite flank (Fig. 5D). Mice vaccinated with ceritinib-treated R80 cells exhibited a delay in tumor growth when compared to non-vaccinated mice (Fig. 5E, S8). These results suggest that R80 cells are indeed immunogenic upon ALK inhibition with ceritinib.

**Fig. 6:**
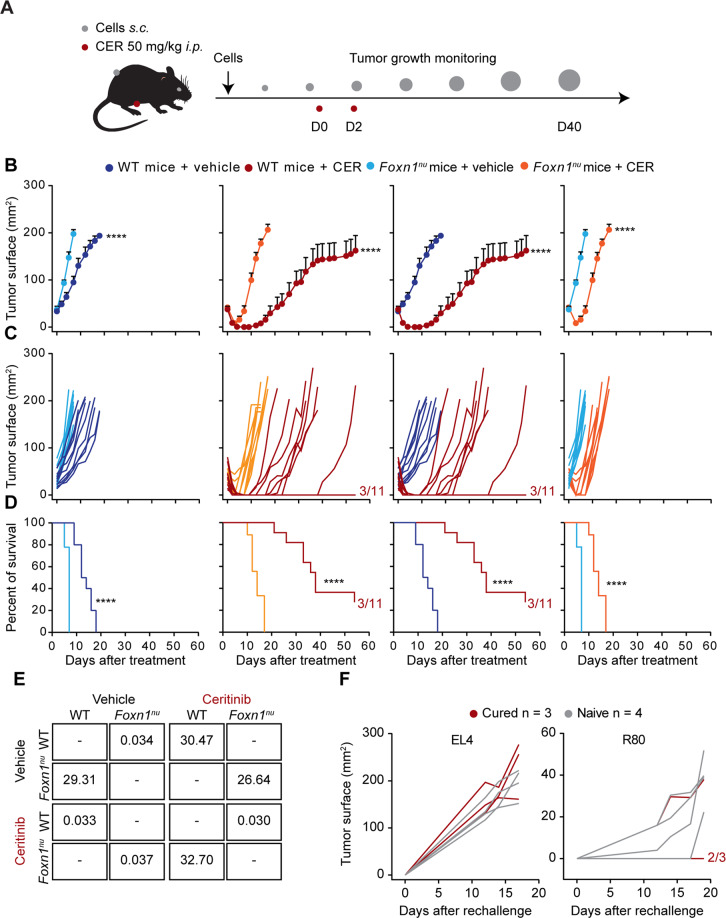
Immune-dependent effect of ceritinib in ALK-driven lymphoma. One million NPM1-ALK^+^ R80 cells were injected into the right flank of C57BL/6 or *Foxn1*^*nu*^ mice. When tumors reached a surface of 30–35 mm^2^ mice were randomized and treated with ceritinib (CER, 50 mg/Kg i.p.) or vehicle control on the day of the randomization (d0) and 2 days later (d2) (**A**). Tumor surface is represented as mean ± SEM of mice belonging to the same group (**B**) or for single mice (**C**). Survival is depicted in **D**. Hazard ratio (Mantel–Haenszel) relative to the comparison between survivals of immunocompetent vs immunodeficient mice or vehicle-treated vs ceritinib-treated is shown in **E**. Vehicle immunocompetent *n* = 10; vehicle immunodeficient *n* = 9; CER immunocompetent *n* = 11; CER immunodeficient *n* = 9. tumor growth curves were compared using a type II ANOVA test, while statistical significance of survival was calculated using the Log-rank test. ****p < 0.0001. Cured mice (*n* = 3) and naïve controls (*n* = 4) were challenged with 1 × 10^6^ R80 into the left flank and 5 × 10^5^ of unrelated lymphoma cells, EL4, into the right flank. Tumor growths of EL4 and R80 lymphomas are shown in **F**.

**Fig. 7:**
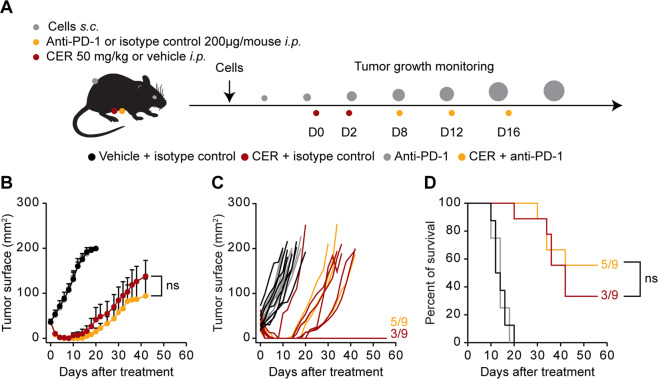
In vivo combination of ceritinib and anti-PD-1. One million of murine NPM1-ALK^+^ R80 cells were injected into the right flank of syngeneic mice (C57BL/6). When tumors reached a surface of 30–35 mm^2^ mice were randomized and treated with ceritinib (CER, 50 mg/Kg, i.p.) or vehicle control on the day of the randomization (d0) and 2 days later (d2). Then, on d8, d12, d16 mice were injected i.p. with 200 μg of anti-PD-1 or isotype control antibodies (**A**). Tumor surface is represented as mean ± SEM of mice belonging to the same group (**B**) or for single mice (**C**). Survival is depicted in **D**. Vehicle + isotype *n* = 8; vehicle + anti-PD-1 *n* = 8; CER + isotype *n* = 9; CER + anti-PD-1 *n* = 9. Comparison between tumor growth curves was assessed using a type II ANOVA test, while statistical significance of survival data was calculated using the Log-rank test. ns non-significant.

Next, we established subcutaneous R80 lymphomas on immunocompetent C57BL/6 mice or immunodeficient *Foxn1*^*nu*^ mice (which are athymic and hence lack thymus-derived T lymphocytes) and treated them with ceritinib or vehicle-only (Fig. 6A). Of note, in this system, vehicle-treated R80 lymphomas developed more quickly on immunodeficient than on immunocompetent mice, suggesting that they are under natural (therapy-independent) immunosurveillance. Ceritinib-treated tumors regressed in a transient fashion, both in immunocompetent and in immunodeficient hosts (Fig. 6B, C). After approximately 10 days, the majority of tumors grew back, but 3 out of 11 mice were permanently (≥60 days) cured in immunocompetent hosts (Fig. 6B, C, E). This is in sharp contrast with the effects of ceritinib observed against R80 lymphomas growing on immunodeficient mice, which all relapsed rather quickly in less than 10 days (Fig. 6B, C, E). These T-lymphocyte-dependent ceritinib effects also influenced the survival of R80 lymphoma-bearing mice. In immunocompetent R80 lymphoma-bearing mice, ceritinib extended median survival by ~20 days, while this interval was reduced to ~10 days in immunodeficient mice (Fig. 6D). When cured mice (which were all immunocompetent) were re-inoculated with R80 tumor cells, 2 out of 3 exhibited a long-term protection. In contrast, naïve mice always (in this experiment 4 out of 4 mice) allowed for R80 lymphoma cells to generate subcutaneous tumors. Antigenically unrelated EL4 lymphoma cells indistinguishably formed tumors in naïve mice and animals ridden from R80 lymphomas (Fig. 6F). Of note, repeated injection of a PD-1-blocking antibody failed to significantly improve tumor growth reduction, but tended to enhance the frequency of long-term (≥ 60 days) cures, which increased from 3 out of 9 cases in R80 lymphoma bearing C57BL/6 mice treated with ceritinib plus isotype control antibody to 5 out of 9 cases in mice treated with ceritinib plus anti-PD-1 antibody (Fig. 7).

We conclude from these results that ALK inhibition by ceritinib can result in therapeutically relevant ICD induction.

## Discussion

Immunogenic cell death (ICD) has mostly been characterized in the context of cytotoxic agents that non-specifically target general cellular functions such as DNA-to-RNA transcription or microtubular polymerization. Thus, historically, anthracyclines and oxaliplatin have been listed among the first ICD inducers [13, 45]. The most recent clinically approved ICD inducers are lurbinectedin, an inhibitor of transcription that was FDA-approved for the treatment of small-cell lung cancer in June 2020 [46], as well as an anti-BCMA antibody conjugated to a microtubular poison (belantamab mafodotin) that was FDA-approved in August 2020 for the treatment of relapsed or refractory multiple myeloma [47]. Our recent work suggests that specific tyrosine kinase inhibitors (TKIs) may have ICD-inducing effects as well. Indeed, a screening initially performed on osteosarcoma U2OS cells revealed that several TKIs, including crizotinib, used at concentration ≥10 µM could elicit the stigmata of ICD in vitro. Since U2OS cells lack constitutive activation of ALK, this effect is clearly off-target and may be explained by the simultaneous inhibition of several tyrosine kinases by high crizotinib doses. Nevertheless, these observations created the precedent that TKIs can induce ICD, though through off-target effects [35].

In the present paper, we characterized on-target effects of crizotinib by specifically evaluating its effects on ALK-positive anaplastic large cell lymphoma (ALK^+^ ALCL) (which depends on ALK as a major oncogenic and trophic factor). Specificity was assured by several parallel strategies, namely by (i) reducing the concentration of crizotinib to lower doses (≤5 µM) that do not affect ALK-independent lymphomas, (ii) replacing crizotinib by ceritinib, which has a higher potency and specificity for ALK [48], (iii) assuring that genetic inhibition (inducible knockdown) of ALK mirrored the effects of its pharmacological interference, (iv) evaluating crizotinib effects on cells that had become resistant to this inhibitor due to mutation of ALK kinase domain. Of note, results obtained by these approaches plead in favor of specific ALK inhibition inducing ICD. Both human and mouse ALK^+^ ALCL cells displayed the hallmarks of ICD (CALR exposure, ATP release, HMGB1 exodus). Moreover, vaccination of mice with ceritinib-treated ALK-dependent ALCL was able to restrain the progression of the same lymphoma inoculated 2 weeks later.

In vivo treatment of ALK^+^ ALCL bearing mice with ceritinib showed that ALK inhibition acted in two complementary ways. First, ceritinib clearly mediated a therapeutic effect that was independent of adaptive T-cell responses and hence significantly reduced the volume of established lymphomas irrespective of the host immune status. Second, tumor shrinkage was followed by relapse, which occurred more frequently (*p* < 0.05, Chi square test) in immunodeficient (9 out of 9) mice (Fig. 6) than in immunocompetent hosts (14 out of 20, Figs. 6 and 7), especially when combined with PD-1 blockade (5 out of 9, Fig. 7). Hence, 30% of immunocompetent mice could be cured from ALK^+^ ALCL by ceritinib (Figs. 6 and 7), and addition of PD-1 blockade (Fig. 7) lead to an increase of this percentage to 55% (*p* < 0.05, Chi square test for data pooled from Figs. 6 and 7). These results strongly indicate that the cell-autonomous effects of ALK inhibitors are complemented by an additional, immune-mediated mechanism of tumor control.

The last decades witnessed great progresses in the treatment of hematological cancers, with a transition from chemotherapeutic regimes hitting all proliferating cells to drugs targeting tumor-specific survival pathways, such as tyrosine kinase inhibitors. Although this shift has led to a spectacular amelioration of cure rates, there is still room for improvement. Recent developments plead in favor of the adoption of immunological concepts to blood cancers. For example, PD-1 blockade showed great clinical success in relapsed or refractory Hodgkin lymphoma [49, 50]. Another promising immunotherapeutic approach is vaccination. In chronic myeloid leukemia patients, remissions have been achieved after vaccination with BCR-ABL-derived peptides [51]. Concerning ALK^+^ ALCL, several studies unequivocally demonstrated that patients develop a specific antitumor immune response. Pulford and collaborators showed the presence of autoantibodies against NPM1-ALK in ALCL patients [52], and higher antibody titers were correlated with improved overall and progression-free survival, as well as reduced relapse rates [53]. Whether such anti- NPM1-ALK antibodies are mere signs of an immune response or true immune effectors, however, has not been clarified, and ICD-related immune responses are mostly mediated by CD8^+^ (not CD4^+^) T cells [54, 55]. Indeed, ALK-specific CD8^+^ T cells occur naturally in healthy subjects and ALCL patients. However, the former show a naïve phenotype, while the latter show an effector or memory phenotype, suggesting a relatively recent activation [56]. Altogether, these observations indicate that immunotherapy might be a therapeutic option for ALK^+^ ALCL. Indeed, two case reports demonstrated that, in relapsed ALK^+^ ALCL patients, PD-1 blockade led to complete and long-lasting remissions [57, 58]. In addition, preclinical data demonstrated that vaccination with DNA plasmids encoding for the cytoplasmic domain of ALK successfully prevented the occurrence of systemic ALK^+^ ALCL [59].

The present work demonstrates that TKIs designed to inhibit ALK oncogenic kinase induce hallmarks of ICD in ALK-dependent tumors, through a rigorous on-target effect. These effects are also reflected by immune responses against ALK-induced anti-lymphoma in a preclinical model. Hence, the successful treatment of ALCL most likely relies, at least in part, on improved immunosurveillance. Future studies must explore the clinical utility of combining ALK inhibitors with immunotherapeutic strategies for the definitive cure of ALK^+^ ALCL.

## Material and methods

### Cell lines and culture conditions

Human NPM1-ALK-positive (NPM1-ALK^+^) anaplastic large cell lymphoma (ALCL) SU-DHL-1 cell line was purchased from the German Collection of Microorganisms and Cell Cultures (DSMZ, Braunschweig, Germany). Human NPM1-ALK^+^ ALCL SUP-M2 and NPM1-ALK-negative (NPM1-ALK^-^) ALCL MAC-1 and FE-PD cell lines were kindly provided by Dr. Meggetto (Centre de Recherches en Cancérologie de Toulouse, France); human NPM1-ALK^+^ ALCL SUP-M2 cell line crizotinib-resistant (SUP-M2CR03) [40] by Dr. Ceccon and Prof. Gambacorti-Passerini (Università degli Studi di Milano-Bicocca, Italy) and human NPM1-ALK^+^ ALCL SU-DHL-1 TTA [39] cell line by Dr. Ducray (University of Cambridge, United Kingdom). Prof. Chiarle (Boston Children’s Hospital, Massachusetts, USA) and Dr. Poggio (Uniklinik Freiburg, Germany) provided the murine NPM1-ALK^+^ cell line R80. Cells were cultured in RPMI 1640 supplemented with 100 units/mL of penicillin, 100 µg/mL of streptomycin (Pen/Strep) and 10% fetal bovine serum (FBS) or 10% tetracycline-free FBS (PAN-Biotech, Aidenbach, Germany) (for SU-DHL-1 TTA cell line). All cell lines were cultured at 37 °C and 5% CO_2_ and routinely checked for mycoplasma contamination. All media and supplements were purchased from Gibco (Waltham, Massachusetts, USA).

### Cell treatments

ALCL cells were harvested, seeded at 2.5–5 × 10^5^/mL and concurrently treated with pharmacological inhibitors. Inhibitors used throughout this study were: crizotinib (CRIZ; Sigma-Aldrich, St. Louis, Missouri, USA), ceritinib (CER; Selleck Chemicals, Houston, Texas, USA), BEZ235 (BEZ; Selleck Chemicals), BKM120 (BKM; Selleck Chemicals), PD0325901 (PD; Sigma-Aldrich), stattic (Selleck Chemicals), U73122 (Tocris, Bristol, UK), BYL719 (BYL; Selleck Chemicals), XL147 (XL; Selleck Chemicals), and Q-VD-OPh (Calbiochem, San Diego, California, USA). Mitoxantrone (MTX) was purchased from Sigma-Aldrich. SU-DHL-1 TTA were treated with 1 μg/mL of doxycycline (Sigma-Aldrich).

### Immunogenic cell death assays

#### Cell death analysis

Cell death was assessed by flow cytometry employing the following fluorescent dyes: 3,3-dihexyloxacarbocyanine iodide (DiOC_6_(3), 20 nM; Thermo Fisher Scientific, Waltham, Massachusetts, USA) selective for mitochondria of live cells; 4′,6-diamidino-2-phenylindole (DAPI; 1 µg/mL, Invitrogen, Carlsbad, California, USA) incorporated by dead cells after cell membrane rupture. After treatment, cells were incubated with DiOC_6_(3) and DAPI diluted in medium, for 30 min at 37 °C/5% CO_2_ [60, 61] and analyzed by means of a MACSQuant flow cytometer (Miltenyi Biotec, Bergisch Gladbach, Germany). Data were analyzed with FlowJo software (Ashland, Oregon, USA).

#### Calreticulin (CALR) exposure

Following treatments, cells were transferred to a 96-well plate and washed twice with 150 µl/well of cold (4 °C) PBS. Then, they were resuspended in 50 µl/well of anti-CALR antibody (Abcam, Cambridge, UK) diluted in PBS supplemented with 1% bovine serum albumin (BSA) and incubated for 45 min on ice. Afterwards, cells were washed twice with 150 µl/well of cold PBS and resuspended in 50 µl/well of goat anti-rabbit IgG-Alexa Fluor 488 or -Alexa Fluor 647 (Invitrogen) diluted in PBS/1% BSA. After 30 min of incubation on ice and two additional washing steps, cells were resuspended in 120 µl/well of cold PBS supplemented with DAPI at 1 µg/mL [61, 62]. Cells were analyzed by means of a MACSQuant flow cytometer and data were analyzed with FlowJo software.

#### Adenosine triphosphate (ATP) secretion

ATP secreted in the extracellular milieu was quantified using the ENLITEN ATP assay (Promega, Madison, Wisconsin, USA). Cell supernatants were collected and cell debris removed by centrifugation at 1000 *g* for 5 min. Then, 20 µl of supernatants were transferred to an opaque-walled 96-well plate together with ATP diluted at 10^−7^ M, 10^−8^ M, 10^−9^ M, 10^−10^ M, and 10^−11^ M as standards and water (blank). Afterwards, 100 µl of rLuciferase/Luciferin (rL/L) reagent were added to supernatants and standards and emitted bioluminescence was measured with a plate reading luminometer (Victor, PerkinElmer, Waltham, Massachusetts, USA). ATP concentrations were calculated using four-parameter logistics.

#### High–Mobility Group Box 1 (HMGB1) release

HMGB1 released in the extracellular environment after treatment was quantified using a sandwich ELISA (IBL International, Hamburg, Germany) following manufacturer’s instructions. Briefly, supernatant was collected and cell debris were removed by centrifugation at 1000 *g* for 5 min. Then, 10 µl of supernatants or HMGB1 standards were added to the microtiter plate together with 100 µl/well of “diluent buffer”. After 20–24 h of incubation at 37 °C and five washing steps with 400 µl/well of “washing buffer”, 100 µl/well of “enzyme conjugate” were added and the plate was incubated for 2 h at room temperature. After five additional washing steps, 100 µl/well of “color solution” were added and incubated for 30 min at room temperature. Color development was stopped with 100 µl/well of “stop solution” and absorbance at 450 nm was measured using a spectrophotometer (Victor, PerkinElmer). Absorbance values of each standard were plotted against their known concentration and unknown HMGB1 concentrations were calculated using four-parameter logistics [63, 64].

#### Interferon beta 1 (IFNB1) and C–X–C motif chemokine 10 (CXCL10) upregulation

Following treatment, total RNA was extracted using the Rneasy plus mini kit (Qiagen, Hilden, Germany) following manufacturer’s instruction. Genomic DNA was digested during RNA purification using the RNase-Free DNase Set (Qiagen) according to manufacturer’s guidance. Next, total RNA was reverse transcribed to cDNA using SuperScript™ IV VILO™ Master Mix (Thermo Fisher Scientific). Briefly, 1 to 2.5 µg of RNA were diluted in 16 µl of nuclease-free water and 4 µl of SuperScript™ IV VILO™ Master Mix were added. Reverse transcription was performed under the following conditions: primer annealing at 25 °C for 10 min, reverse transcription at 50 °C for 10 min, and heat inactivation at 85 °C for 5 min. Thereafter, *IFNB1*, *CXCL10*, or *GAPDH* were amplified using specific TaqMan Gene Expression assays (Thermo Fisher Scientific) using the TaqMan Fast Advanced Master Mix (Thermo Fisher Scientific) on the StepOnePlus Real-Time PCR System (Applied Biosystems, Waltham, Massachusetts, USA). Running conditions were 50 °C for 2 min, 95 °C for 2 min followed by 40 cycle of target gene amplification (95 °C for 1 s and 60 °C for 20 s). Finally, threshold cycle (*C*_*t*_) values of genes of interest were subtracted to *C*_*t*_ values of the housekeeping gene, *GAPDH*, generating Δcts; fold change of expression between crizotinib-treated and vehicle-treated samples was calculated using the formula: 2^(-ΔΔCt)^.

#### Quinacrine staining

Cells were transferred to a 96-well plate and washed twice with 150 µl/well of “Krebs solution” (125 mM NaCl, 5 mM KCl, 1 mM MgSO_4_, 0.7 mM KH_2_PO_4_, 2 mM CaCl_2_, 6 mM Glucose, 25 mM 4-(2-hydroxyethyl)−1-piperazineethanesulfonic acid or HEPES, H_2_O). Afterwards, they were resuspended in 100 µl/well of quinacrine (Sigma-Aldrich) diluted in “Krebs solution” at 2 μM and incubated for 30 min at 37 °C/5% CO_2_. Then, after two more washing steps, cells were resuspended in 120 μl/well of “Krebs solution” supplemented with 1 µg/mL of DAPI and cells were analyzed by means of a MACSQuant flow cytometer [43, 65]. Data were analyzed with FlowJo software.

### Immunoblotting

After treatment, cells were lysed in 150 µl of RIPA buffer (Sigma-Aldrich) for 45 min on ice. After removing cell debris, by centrifugation at 14,000 rpm for 15 min, protein extracts were loaded onto 4–12% polyacrylamide gel (Invitrogen) and then transferred to a 0.2 µm nitrocellulose membrane (Bio-Rad, Hercules, California, USA). Unspecific binding sites were saturated incubating membranes with Tris-buffered saline (TBS) supplemented with 0.05% Tween 20 (TBST) and 5% non-fat powdered milk or BSA. Thereafter, membranes were incubated with primary antibodies diluted in TBST/5% BSA for 16–18 h at 4 °C. Antibody binding was revealed using a suitable secondary antibody coupled to horseradish peroxidase conjugates (HRP) (Southern Biotech, Birmingham, Alabama, USA) diluted in TBST/5% milk and incubated for 1 h, followed by chemiluminescence-based detection (Amersham, Little Chalfont, UK). Images were acquired using the ImageQuant LAS 4000 software-assisted imager (GE Healthcare, Chicago, Illinois, USA). Quantification was performed with ImageJ software.

### Antibodies

Anti-CALR (ab2907) antibody was purchased from Abcam. Anti-ALK (3633), -phospho-Akt (9271), -total Akt (9272), -caspase-3 (9662), -caspase-8 (9746), -phospho-eIF2α (9721), -total eIF2α (9722) -phospho- ERK1/2 (4370), -total ERK1/2 (4695) were purchased from Cell Signaling Technology (Danvers, Massachusetts, USA). Anti-CD11c coupled to PE-Vio770 (clone N418) was purchased from Miltenyi Biotec, whereas anti-CD86-Alexa Fluor 647 (clone GL-1) and anti-Ia/Ie-APC (cloneM5/114.15.2) from Biolegend (San Diego, California, USA). Anti-rabbit (4050-05) and anti-mouse (1031-05) secondary antibodies conjugated to horseradish peroxidase (HRP) were purchased from Southern Biotech; anti-rabbit secondary antibodies coupled to Alexa Fluor 488 (A-11034) or Alexa Fluor 647 (A-32733) came from Invitrogen. Antibodies for in vivo purpose: anti-PD-1 (BE0273, clone 29 F.1A12) and rat IgG2a anti-trinitrophenol isotype control (BE0089, clone LTF-2) were purchased from BioXcell (Lebanon, New Hampshire, USA).

### Phagocytosis and bone marrow-derived dendritic cell maturation assays

#### BMDCs

Bone marrow-derived dendritic cells (BMDCs) were generated from bone marrow of femurs and tibias of C57BL/6 mice. First, bone marrows were collected purging bones with PBS supplemented with Pen/Strep and 2% FBS. Then, red blood cells were lysed with 5 mL of lysis buffer (BioLegend). After filtration, through a 70 μm cell strainer, and washing, cells were resuspended in RPMI 1640 medium supplemented with Pen/Strep, 10 mM HEPES, 1% Non-Essential Amino Acids Solutions, 10% FBS, 50 nM β-mercaptoethanol, 20 ng/ml of recombinant granulocyte-macrophage colony-stimulating factor (GM-CSF) and 20 ng/ml of FMS-like tyrosine kinase 3 ligand (FLT3L) (Peprotech, Rocky Hill, New Jersey, USA) (complete medium) and seeded into 6-well plates at 7.5 × 10^5^/mL. After three days of culture, 1 mL of complete medium was added to each well and after three additional days, half of the culture medium was replaced. On day 7 BMDCs were harvested and resuspended at 2 × 10^5^/mL for co-culture.

#### Tumor cells

R80 cells were stained with 0.5 μM of the CellTracker green 5-chloromethylfluorescein diacetate (CMFDA; Thermo Fisher Scientific) (for the phagocytosis assay) or left unstained (for the BMDC maturation assay). Briefly, cells were harvested and resuspended at 2 × 10^6^/mL in serum-free medium and incubated with CMFDA for 15 min at 37 °C/5% CO_2_. Free CMFDA was neutralized adding an equal volume of FBS. After centrifugation, cells were resuspended in serum-containing medium and incubated for 30 min at 37 °C/5% CO_2_. Afterwards, R80 cells were treated and subsequently co-cultured with BMDCs at 1:4 ratio (BMDCs:murine tumor cells).

#### Co-culture

After 4 h, cells were detached with a cell scraper, and cell suspensions were transferred to 96-well plates. After washing with 150 μl/well of cold (4 °C) PBS, cells were stained with 50 μl/well of anti-CD11c antibody coupled to the PE-Vio770 fluorophore diluted in PBS/1% BSA. After 30 min of incubation on ice, cells were washed and fixed with 3.7% formaldehyde diluted in PBS. Finally, cells were analyzed by means of a MACSQuant flow cytometer, and data analyzed with FlowJo software. Percentage of phagocytized cells equaled to the percentage of CMFDA^+^ cells within CD11c^+^ BMDCs (phagocytosis assay) [66]. BMDCs maturation assay was performed after 24 h of co-culture. First, cells were detached with a cell scraper and transferred to 96-well plates. After washing with 150 μl/well of cold PBS, cells were incubated with 50 μl/well of the LIVE/DEAD fixable violet dead cell stain kit (Invitrogen) for 15 min on ice. After 2 additional washings, cells were stained with fluorescent anti-CD11c, -CD86, and -Ia/Ie antibodies for 30 min on ice. Finally, cells were fixed with 3.7% formaldehyde (diluted in PBS) and fluorescence intensity of single cells was quantified using a MACSQuant flow cytometer. Data were analyzed with FlowJo software. Fold increase of expression was calculated using the median of fluorescence intensity of each marker [67].

### In vivo experiments

#### Animals

Six- to eight-week-old female wild-type C57BL/6 and *Foxn1*^*nu*^ mice were purchased from Envigo (Indianapolis, Indiana, USA) and housed in a pathogen-free, temperature-controlled environment with 12-h day and night cycles. They received water and food ad libitum. Animal experiments were conducted in compliance with the EU Directive 63/2010 and with protocols #18967-2019020612051799v2 and #28893-2021010810535218v2 approved by the local Ethical Committee (“C. Darwin” registered at the French Ministry of Research).

#### Vaccination assay

R80 cells were treated with ceritinib or mitoxantrone to reach 50–70% mortality. After washing with cold (4 °C) PBS, they were resuspended at 10–50 × 10^6^/mL. 100 μl of dying cells were injected subcutaneously (s.c.) into the left flank of immunocompetent C57BL/6 animals. One-two weeks later, 1 × 10^6^ of live R80 tumor cells were injected s.c. into the opposite flank and tumor appearance and growth were monitored. Tumor length and width were measured with a caliper 3 times/week and tumor areas were calculated multiplying length and width. Mice were sacrificed when tumors reached 1.8–2 cm^2^ or if depicting any signs of discomfort. Tumor growth was analyzed with the TumGrowth software package [68], while survival was assessed using the Log-rank test with GraphPad Prism software 9 (San Diego, California, USA).

#### Tumor growth

C57BL/6 and *Foxn1*^*nu*^ mice were injected with 1 × 10^6^ R80 tumor cells s.c. into the right flank. When tumors reached a surface of approximately 30–35 mm^2^, mice were randomized based on tumor size and treated. No blinding was done. Ceritinib, diluted in 76% physiological solution, 10% Tween80, 10% polyethylene glycol (PEG) 400, and 4% DMSO, was administered intraperitoneal (i.p.) twice (on the day of the randomization and 2 days later) at 50 mg/kg. 200 μg of anti-PD-1 or isotype control, diluted in 200 μl of PBS, were administered i.p, 6, 10, and 14 days after the last injection of ceritinib. Tumor length and width were assessed with a caliper 3 times/week and tumor areas were calculated multiplying length and width. Mice were sacrificed when tumors reached 1.8–2 cm^2^ or if depicting any signs of discomfort. Tumor growth was analyzed with the TumGrowth software package [68], while survival was assessed using the Log-rank test with GraphPad Prism software 9.

### Statistical analyses

In vitro*-* and ex-vivo-data are presented as individual replicates or as means ± SD of technical replicates of at least three independent experiments as specified in the figure legends. No statistical method was used to choose sample size. Statistical significance was calculated using the two-tailed Student’s *t* test with Welch’s correction (no assumption of equal SDs). Holm–Sidak correction for multiple comparison was applied when using multiple Student’s *t* tests. Statistical analysis was performed using GraphPad Prism software 9.

In vivo*-*tumor growth data are depicted as means ± SEM of mice belonging to the same group. No statistical method was used to choose sample size. Normal distribution of data was verified plotting tumor areas on a quantile–quantile plot. Statistical significance was calculated using the type II ANOVA test using the TumGrowth software package [68] freely available at https://github.com/kroemerlab. Analysis of survival was performed using the Log-rank test with GraphPad Prism software 9 or Chi-square test.

Statistical significance was represented as following: *p < 0.05, **p < 0.01, ***p < 0.001, ****p < 0.0001.

## Supplementary information

Supplemental figure legend

Supplemental figure 1

Supplemental figure 2

Supplemental figure 3

Supplemental figure 4

Supplemental figure 5

Supplemental figure 6

Supplemental figure 7

Supplemental figure 8
