# Supplementary material for: Pharmacological inhibitors of anaplastic lymphoma kinase (ALK) induce immunogenic cell death through on-target effects
Source: Cell Death Dis. 2021 Jul 16;12(8):713. doi: 10.1038/s41419-021-03997-x (PMC8285454; doi:10.1038/s41419-021-03997-x)
Supplement: Supplementary file 1 — Supplemental figure legend [file 41419_2021_3997_MOESM1_ESM.docx]

**Supplemental figure legends**

**Figure S1. Representative gating strategy of cell death assay**

Representative gating strategy of cell death evaluated using the mitochondrial transmembrane potential-sensible dye (DiOC_6_(3)) and DAPI. Representative dot plots for the human NPM1-ALK^+^ SU-DHL-1 (A) and NPM1-ALK^-^ FE-PD (B) are shown.

**Figure S2. Caspase-dependency of cell death induced by crizotinib and ceritinib**

Human NPM1-ALK^+^ SU-DHL-1 (A) and SUP-M2 (B) cells were pre-treated for 30 min with 10 μM of the pan-caspase inhibitor Q-VD-Oph and then, without washing, challenged with increasing concentrations of crizotinib (CRIZ) or ceritinib (CER) for 24 h. Cell death was assessed by flow cytometry using DiOC_6_(3), which permeate only viable mitochondria, and DAPI, excluded by cells with an intact membrane. Percentage of DiOC_6_(3)^-^ DAPI^-^ or DAPI^+^ cells is depicted as mean ± SD of three independent experiments (n=9). Statistical significance was calculated using the Student’s t-test. **p < 0.01, ****p < 0.0001 *vs*. CRIZ or CER treated cells without Q-VD-Oph (DAPI^+^ cells). ###p < 0.001, ####p < 0.0001 *vs*. CRIZ or CER treated cells without Q-VD-Oph (DiOC_6_(3)^-^ DAPI^-^ cells) (A, B).

**Figure S3. Crizotinib- and ceritinib-induced immunogenic cell death in SUP-M2**

NPM1-ALK^+^ SUP-M2 cells were treated with crizotinib (CRIZ) or ceritinib (CER) at the indicated concentrations and calreticulin (CALR) exposed on the cell surface was quantified by flow cytometry using an indirect staining. DAPI was used to exclude permeabilized cells. Representative gating strategy is shown in (A) and percentage of CALR^+^ DAPI^-^ cells is depicted for single replicates (n=3) in (B).

NPM1-ALK^+^ SUP-M2 cells were treated with CRIZ or CER for 24 h and ATP secreted in the extracellular milieu was quantified using a luciferase-dependent assay. Values of single replicates (n=3) are depicted in (C).

NPM1-ALK^+^ SUP-M2 cells were treated with CRIZ or CER for 48 h and high mobility group box 1 (HMGB1) was quantified in the supernatant using a specific ELISA (n=3) (D). One representative out of three independent experiments is shown (B-D).

NPM1-ALK^+^ SUP-M2 cells were treated with 1 μM of CRIZ for 16-18 h. *IFNB1* and *CXCL10* up-regulation was assessed by quantitative PCR using specific fluorescently labeled primer-probe sets. *GAPDH* was used as housekeeping gene. Fold increases of three independent experiments are shown (G).

Statistical significance was calculated using the Student’s t-test. *p < 0.05, **p < 0.01, ***p < 0.001, ****p < 0.0001 *vs.* vehicle-treated cells (B-E).

**Figure S4. ALK downstream pathway analysis**

Human NPM1-ALK^+^ SU-DHL-1 (A) and SUP-M2 (B) cells were treated for 4 h with mTOR/PI3K, PI3K, ERK1/2 or ALK inhibitors (BEZ235, BKM120, PD0325901, crizotinib) at the indicated concentrations. After treatment, proteins were extracted, separated on polyacrylamide gels and transferred to nitrocellulose membranes. Phosphorylation levels of eIF2α, Akt and ERK1/2, together with caspase-3 and -8 cleavage, were evaluated. Actin was used as loading control. The images are representative of one experiment out of n=3.

**Figure S5. Representative gating strategy of quinacrine staining**

Quinacrine propensity to accumulate in ATP-rich vesicles was exploited to quantify the intracellular content of ATP by flow cytometry after 16-18 h of treatment. DAPI was employed as exclusion dye for permeabilized cells. Representative gating strategy used for SU-DHL-1 (A) and SUP-M2 (B) is shown.

**Figure S6. Induction of cell death and ICD-associated alterations by two PI3K inhibitors, BYL719 and XL147**

SU-DHL-1 and SUP-M2 were treated with 0.1 μM, 0.2 μM, 0.5 μM, 1 μM, 2 μM, 5 μM, 10 μM, 20 μM and 50 μM of BYL719 (BYL) or XL147 (XL) and cell death was evaluated using DiOC_6_(3) and DAPI after 24 h. The number of live cells (DiOC_6_(3)^+^ DAPI^-^), normalized to vehicle control, is represented for single replicates (A). Calreticulin exposure was measured by means of indirect fluorescent staining at 18 and 24 h for SU-DHL-1 and SUP-M2 cells, respectively (B). ATP release quantified after 18 h (C) and HMGB1 exodus after 24 h (D). Values (n=3) of one representative experiment out of two are shown (A-D). Numbers indicate µM concentrations. Statistical significance was calculated using the Student’s t-test. *p < 0.05, **p < 0.01, ***p < 0.001, ****p < 0.0001 vs. vehicle-treated cells. N.D., non-detectable.

**Figure S7. ALK inhibition-induced ICD triggers bone-marrow dendritic cell maturation**

Scheme in (A) illustrates dendritic cell maturation assay. Murine tumor cells were treated while bone marrow-derived dendritic cells (BMDCs) were being isolated and differentiated *in vitro* from syngeneic mice. After co-culture, BMDC maturation was assessed by flow cytometry. (B, C) Murine NPM1-ALK^+^ R80 cells were treated with 0.2 μM of crizotinib (CRIZ) or ceritinib (CER), 2 μM of BKM120 (BKM) for 16-18 h or subjected to 1 cycle of freeze-thawing (F/T) and co-cultured with BMDCs for 24 h. BMDC maturation marker (CD86 and MHC-II) were stained using specific fluorescent antibodies. Representative gating strategy is shown in (B). CD86 or MHC-II median of fluorescence intensity (MFI) fold increases are reported in (C) as mean ± SD of n=12 from four independent experiments; *p < 0.05, **p < 0.01, ***p < 0.001, ****p < 0.0001 (Student’s t-test).

**Figure S8. Vaccination assay**

Scheme of vaccination experiment (A). Murine tumor cells were treated *in vitro* to reach 50-70% of mortality before being injected into the left flank of syngeneic mice. One week later, mice were challenged with live cells into the opposite flank and tumor appearance and growth are monitored.

One million of NPM1-ALK^+^ R80 cells, previously treated for 16-18 h with 1 μM of ceritinib (CER) or 2 μM of mitoxantrone (MTX), were injected into the left flank of C57BL/6 mice. After one week, mice were challenged with 1x10^6^ of live R80 cells into the opposite flank. Tumor surface in function of time is represented as mean ± SEM of mice belonging to the same group or for each mouse (E). Survival is also represented in (E). Vehicle n=10, CER n=8 and MTX=10. Comparison between tumor growth curves was assessed using a type II ANOVA test, while the statistical significance of survivals was calculated using the Long-rank test. *p < 0.05, **p < 0.01, ***p < 0.001.
