# Supplementary figures and images for: Pharmacological inhibitors of anaplastic lymphoma kinase (ALK) induce immunogenic cell death through on-target effects
Source: Cell Death Dis. 2021 Jul 16;12(8):713. doi: 10.1038/s41419-021-03997-x (PMC8285454; doi:10.1038/s41419-021-03997-x)

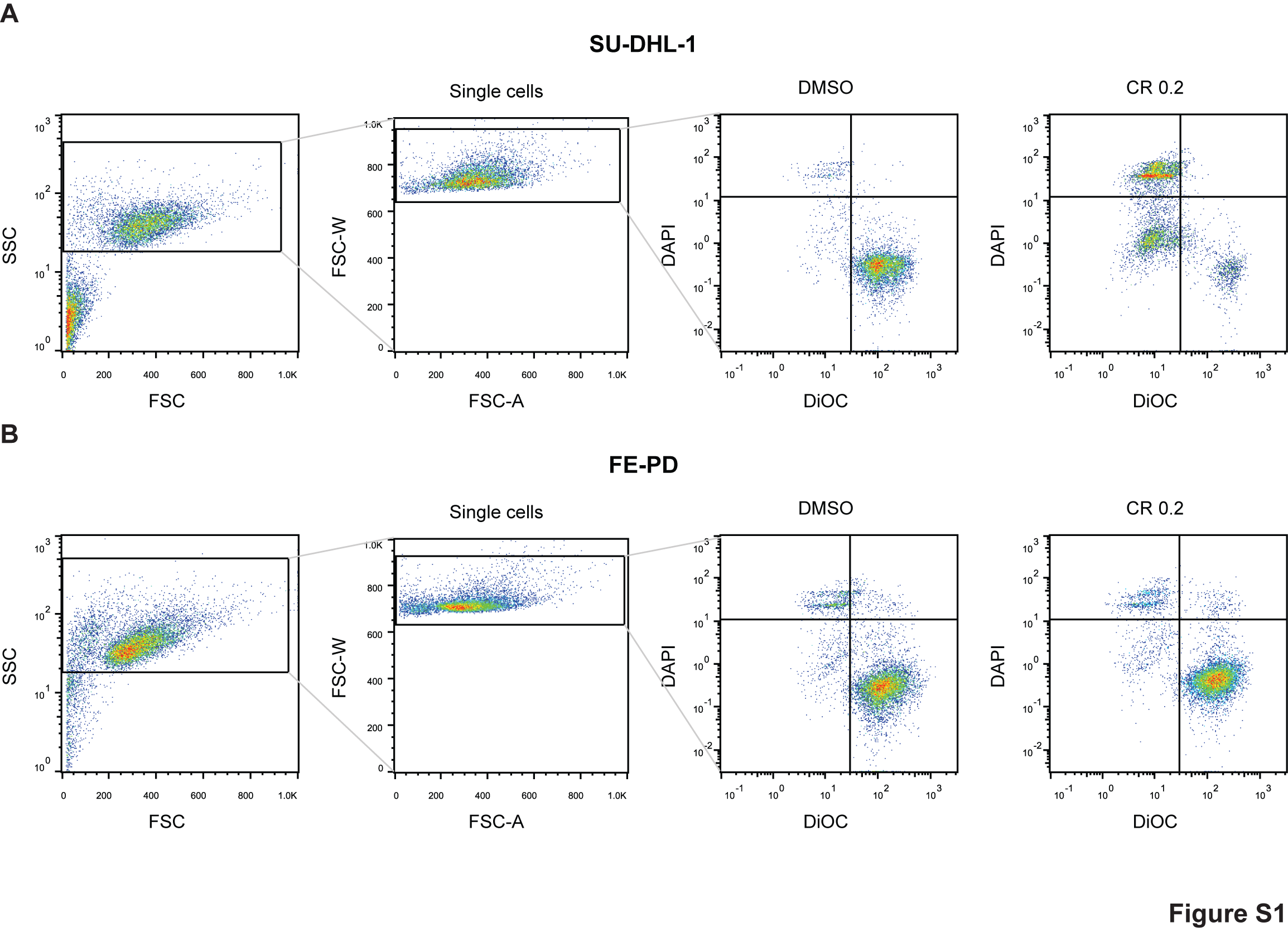

Supplement: Supplementary file 2 — Supplemental figure 1 [file 41419_2021_3997_MOESM2_ESM.png]

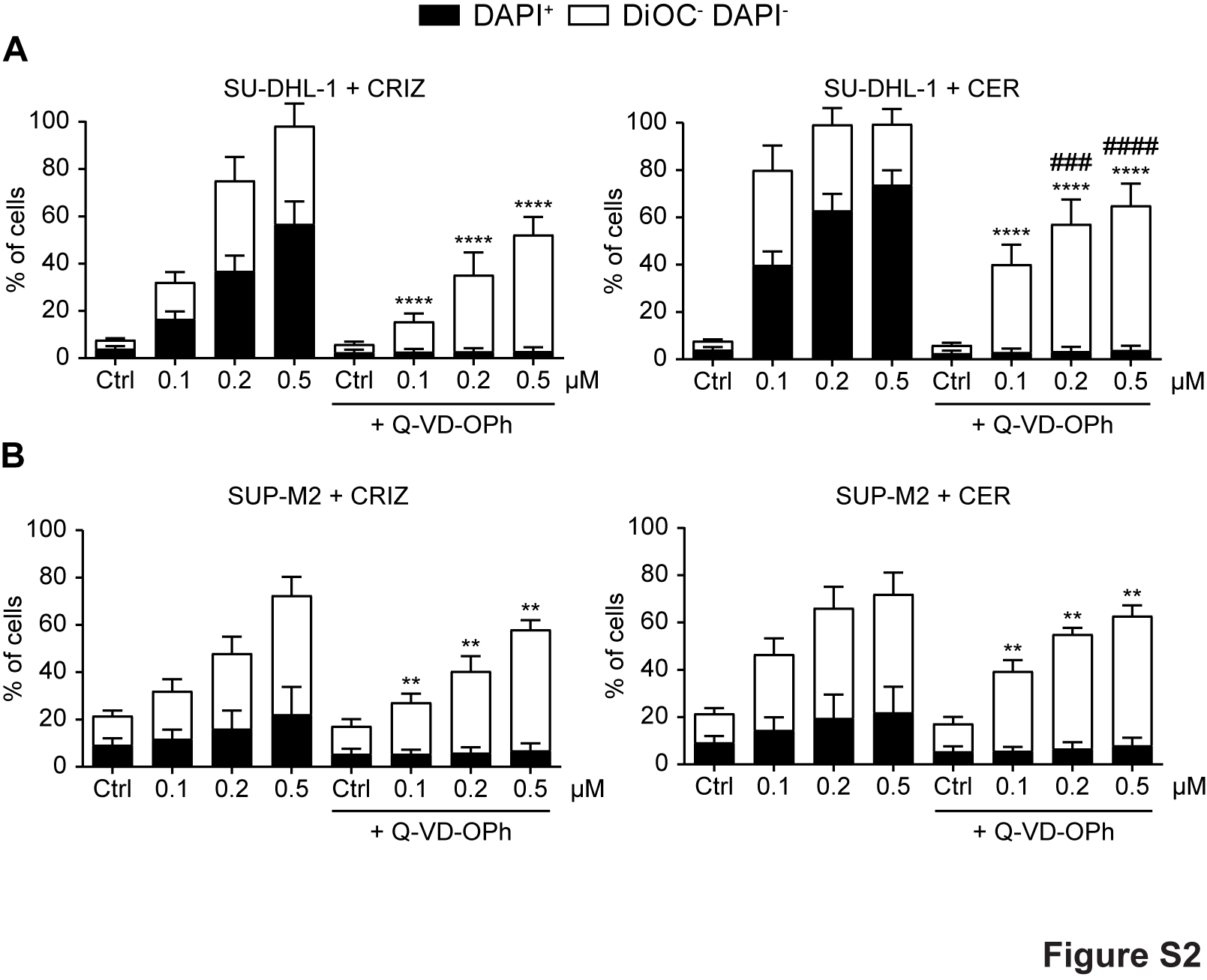

Supplement: Supplementary file 3 — Supplemental figure 2 [file 41419_2021_3997_MOESM3_ESM.png]

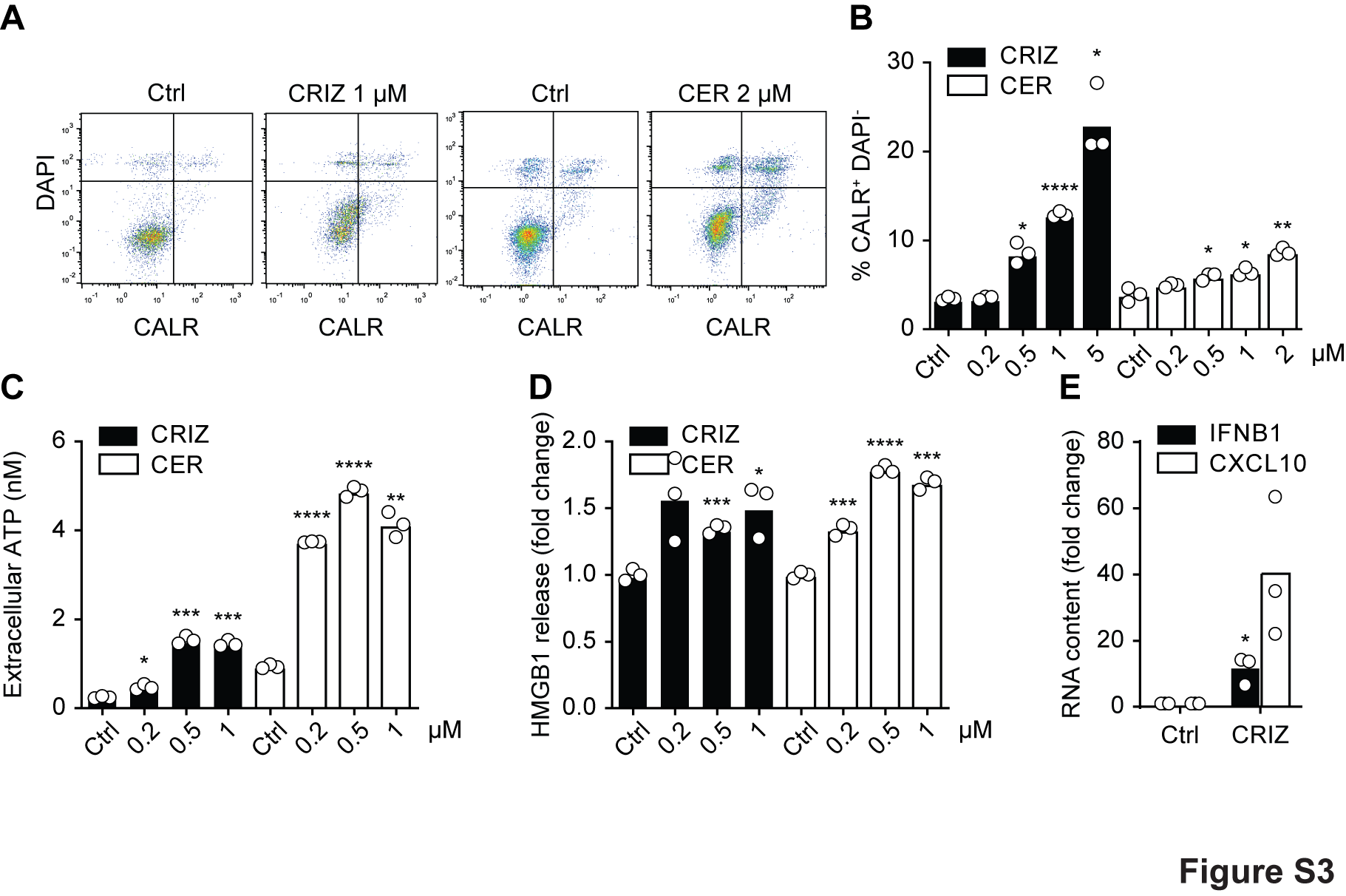

Supplement: Supplementary file 4 — Supplemental figure 3 [file 41419_2021_3997_MOESM4_ESM.png]

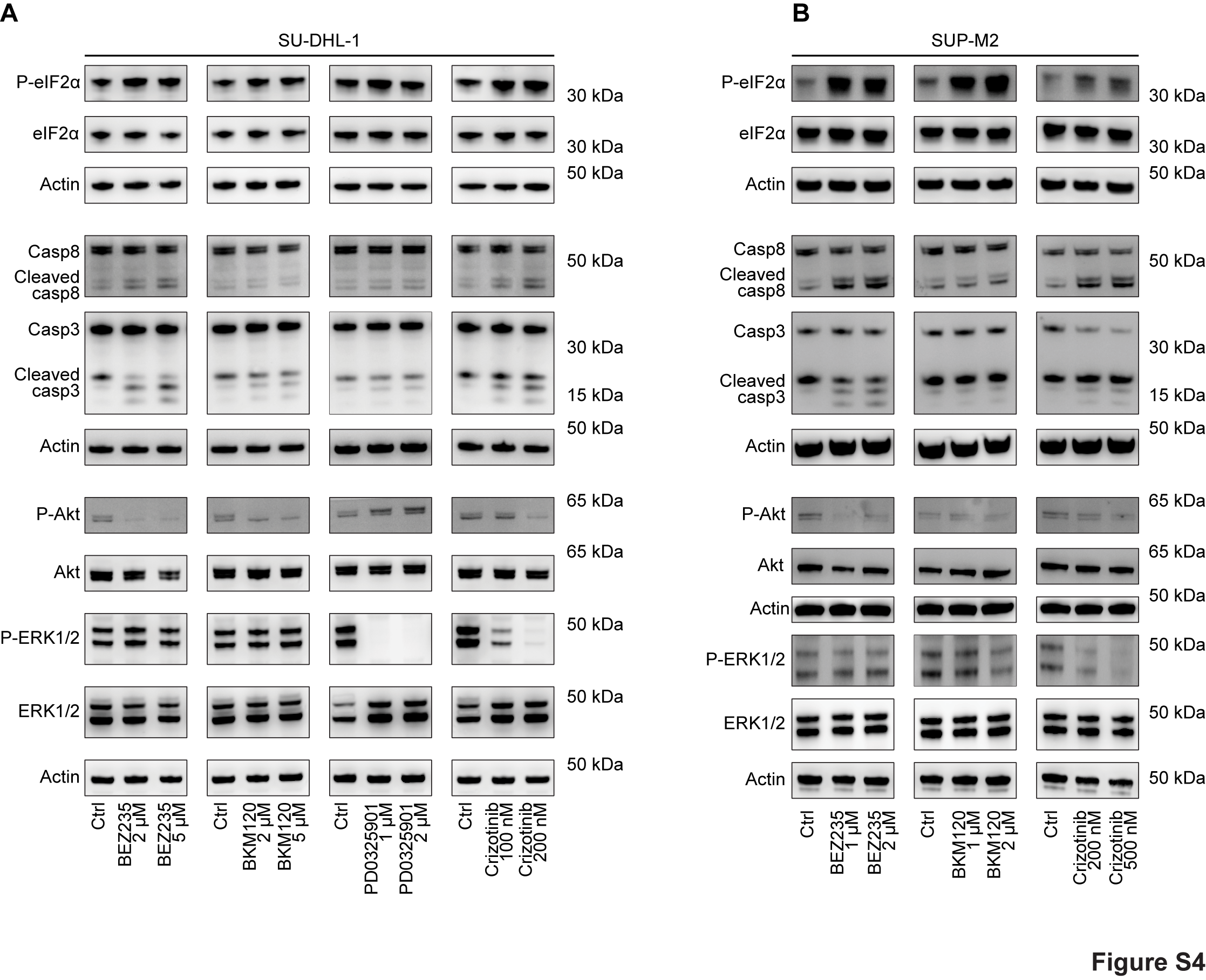

Supplement: Supplementary file 5 — Supplemental figure 4 [file 41419_2021_3997_MOESM5_ESM.png]

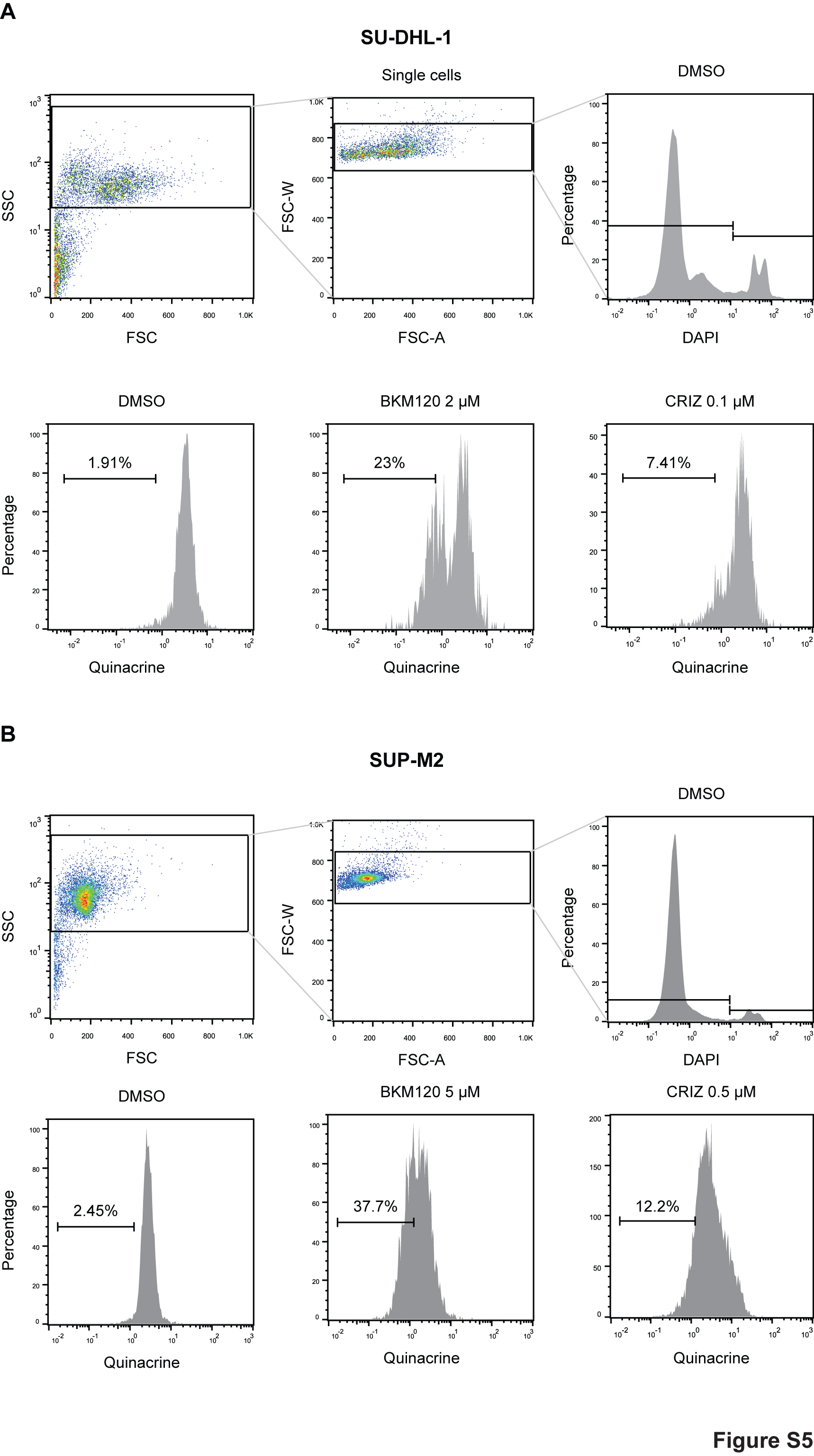

Supplement: Supplementary file 6 — Supplemental figure 5 [file 41419_2021_3997_MOESM6_ESM.png]

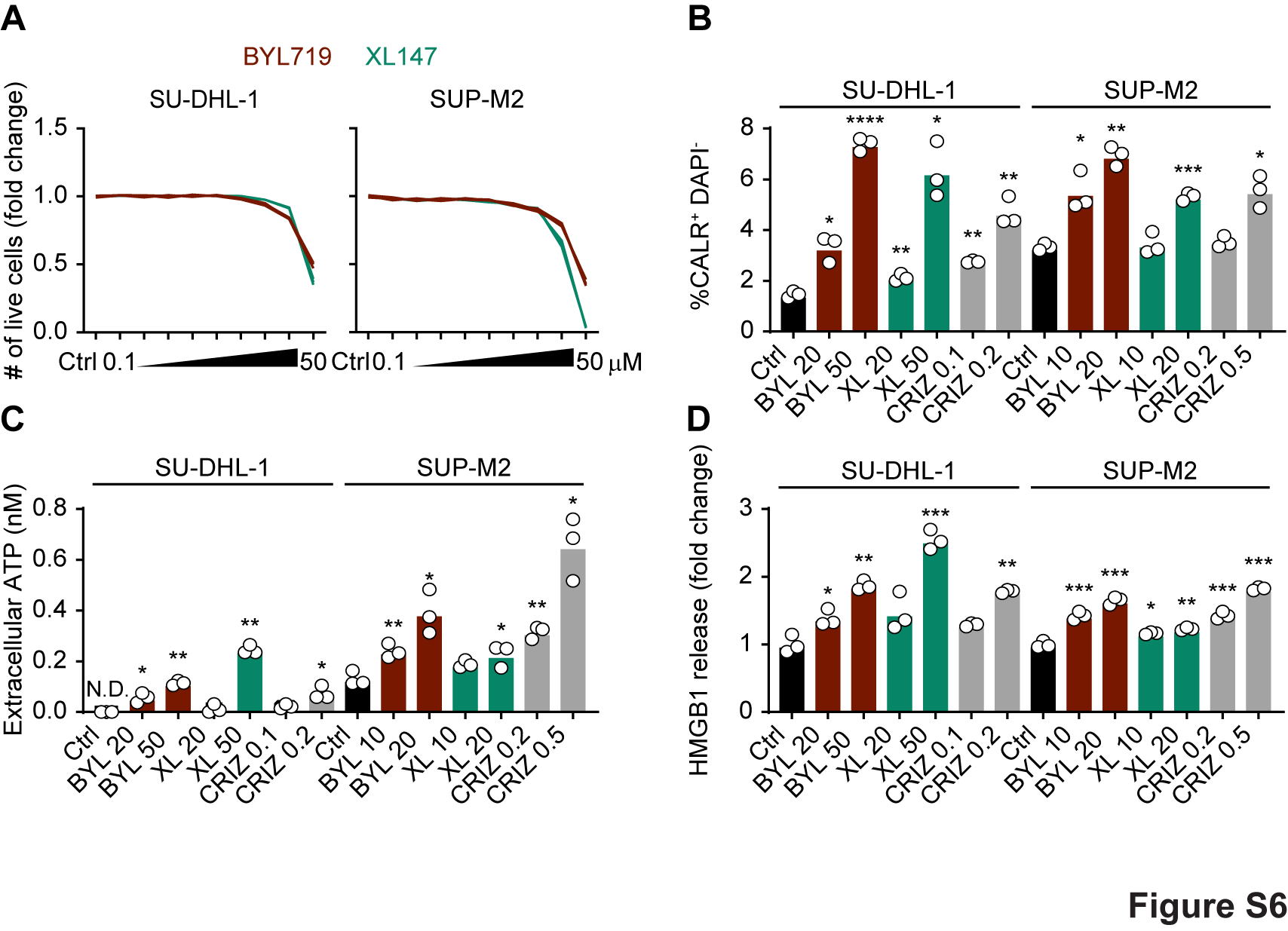

Supplement: Supplementary file 7 — Supplemental figure 6 [file 41419_2021_3997_MOESM7_ESM.png]

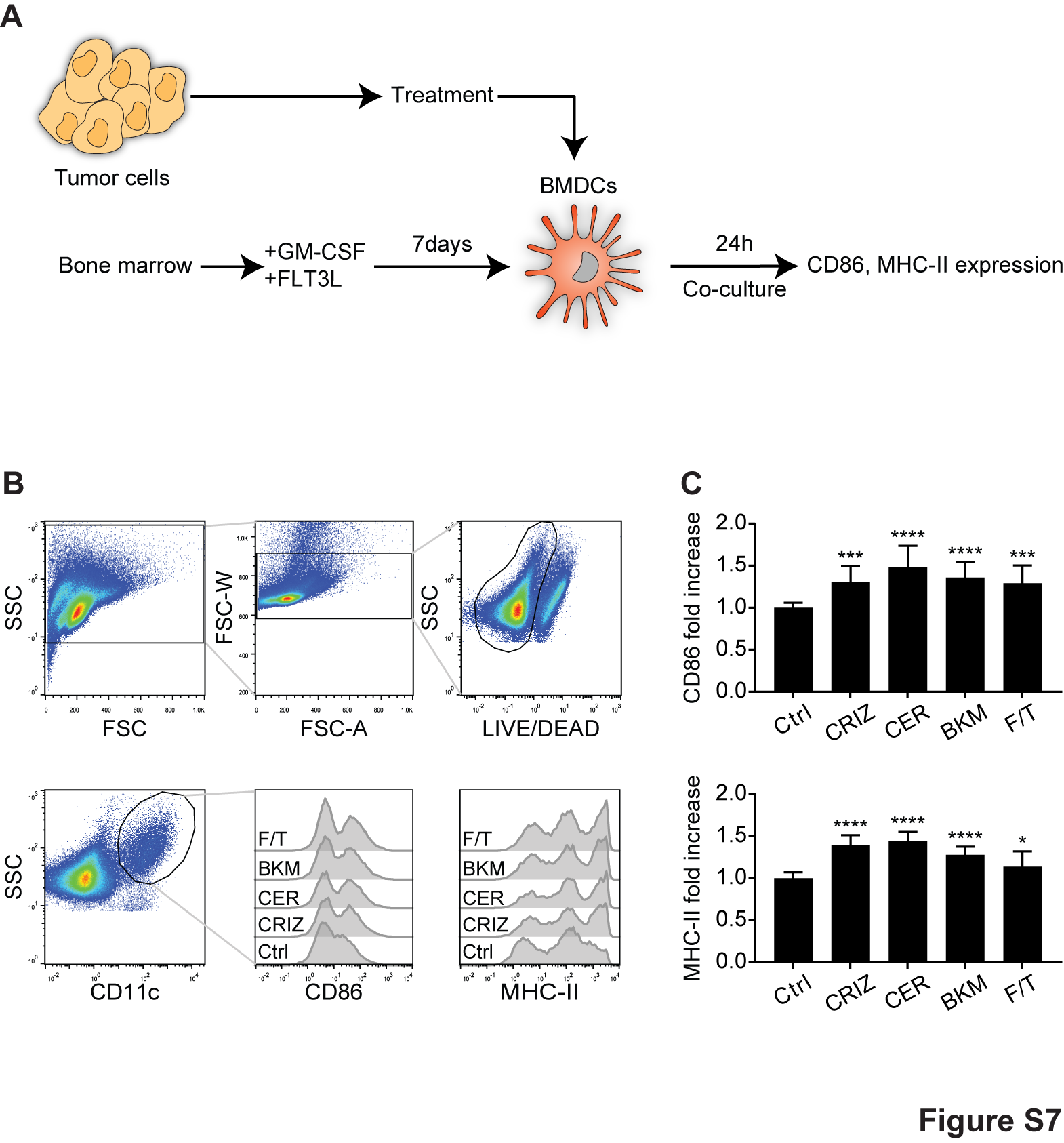

Supplement: Supplementary file 8 — Supplemental figure 7 [file 41419_2021_3997_MOESM8_ESM.png]

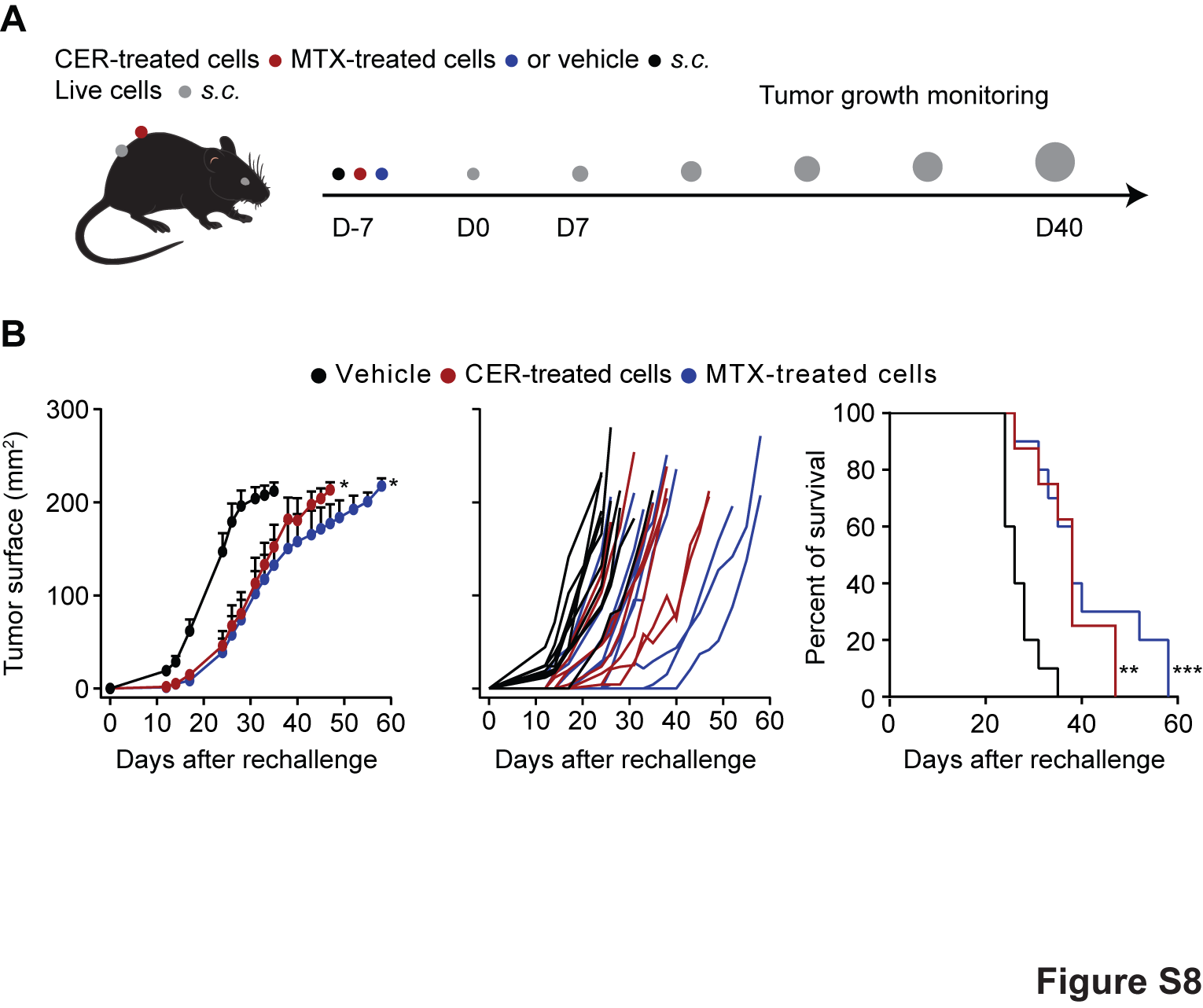

Supplement: Supplementary file 9 — Supplemental figure 8 [file 41419_2021_3997_MOESM9_ESM.png]
